# Recognition motifs rather than phylogenetic origin influence the ability of targeting peptides to import nuclear-encoded recombinant proteins into rice mitochondria

**DOI:** 10.1007/s11248-019-00176-9

**Published:** 2019-10-10

**Authors:** Can Baysal, Ana Pérez-González, Álvaro Eseverri, Xi Jiang, Vicente Medina, Elena Caro, Luis Rubio, Paul Christou, Changfu Zhu

**Affiliations:** 1 Department of Plant Production and Forestry Science, University of Lleida-Agrotecnio Center, Av. Alcalde Rovira Roure, 191, 25198 Lleida, Spain; 2 Centre for Plant Biotechnology and Genomics, Universidad Politécnica de Madrid (UPM) - Instituto Nacional de Investigación y Tecnología Agraria y Alimentaria (INIA), Campus Montegancedo UPM, 28223 Pozuelo de Alarcón, Madrid Spain; 3 ICREA, Catalan Institute for Research and Advanced Studies, Passeig Lluís Companys 23, 08010 Barcelona, Spain

**Keywords:** Subcellular targeting, Mitochondrial pre-sequence, Mitochondrial protein, Protein sorting, Green fluorescent protein

## Abstract

Mitochondria fulfil essential functions in respiration and metabolism as well as regulating stress responses and apoptosis. Most native mitochondrial proteins are encoded by nuclear genes and are imported into mitochondria via one of several receptors that recognize N-terminal signal peptides. The targeting of recombinant proteins to mitochondria therefore requires the presence of an appropriate N-terminal peptide, but little is known about mitochondrial import in monocotyledonous plants such as rice (*Oryza sativa*). To gain insight into this phenomenon, we targeted nuclear-encoded enhanced green fluorescent protein (eGFP) to rice mitochondria using six mitochondrial pre-sequences with diverse phylogenetic origins, and investigated their effectiveness by immunoblot analysis as well as confocal and electron microscopy. We found that the ATPA and COX4 (*Saccharomyces cerevisiae*), SU9 (*Neurospora crassa*), pFA (*Arabidopsis thaliana*) and OsSCSb (*Oryza sativa*) peptides successfully directed most of the eGFP to the mitochondria, whereas the MTS2 peptide (*Nicotiana plumbaginifolia*) showed little or no evidence of targeting ability even though it is a native plant sequence. Our data therefore indicate that the presence of particular recognition motifs may be required for mitochondrial targeting, whereas the phylogenetic origin of the pre-sequences probably does not play a key role in the success of mitochondrial targeting in dedifferentiated rice callus and plants.

**Electronic supplementary material:**

The online version of this article (10.1007/s11248-019-00176-9) contains supplementary material, which is available to authorized users.

## Introduction

The proteins synthesized by eukaryotic cells are targeted to particular subcellular compartments. In the absence of specific targeting signals, nascent proteins accumulate by default in the cytosol (Kim and Hwang 2013). Proteins carrying an N-terminal signal peptide recognized by a signal recognition particle (SRP) on the surface of the endoplasmic reticulum (ER) are co-translationally imported into the secretory pathway, from where they can be routed to various other compartments including the nucleus (Luirink and Sinning 2004; Akopian et al. 2013). Proteins carrying other types of N-terminal or C-terminal peptides are directed post-translationally to peroxisomes, mitochondria or (in plants) to the plastids (Egea et al. 2005). Whereas most organelles are thought to have originated ultimately from the plasma membrane during eukaryote evolution, the mitochondria and plastids are exceptional because they evolved independently from endosymbionts and therefore carry their own genomes (Dolezal et al. 2006). Accordingly, mitochondrial and plastid proteins can be derived either from the organelle genome or the nuclear genome, hence the need for protein import pathways for nuclear-encoded proteins (Chacinska et al. 2009; Endo et al. 2011).

Mitochondria carry out multiple essential functions in the eukaryotic cell, including the generation of ATP by oxidative phosphorylation, the biosynthesis of amino acids and lipids, and the regulation of apoptosis (Rasmusson et al. 2004; Sluse et al. 2006). Approximately 98% of the enzymes and other proteins required for mitochondrial functions are encoded by the nuclear genome and the remainder by the mitochondrial genome (Taylor and Pfanner 2004). Proteins encoded by the nuclear genome must therefore be imported into the mitochondria, and accordingly the pre-proteins carry N-terminal peptides that are recognized by the hydrophobic binding pockets of the receptor Tom20 (Yamamoto et al. 2011). The targeting peptides are cleaved off by mitochondrial peptidases during or after import, yielding the mature protein (Brix et al. 1997; Obita et al. 2003; Taylor and Pfanner 2004; Mukhopadhyay et al. 2006; Wiedemann and Pfanner 2017).

The targeting of recombinant proteins to plant mitochondria could be useful when the aim is to modulate a mitochondrial function such as energy generation, iron–sulfur cluster assembly, developmental signals, or responses to biotic and abiotic stress (Pierrel et al. 2007; Atkin and Macherel 2009). Furthermore, the low-oxygen mitochondrial environment is ideal for metabolic engineering with oxygen-sensitive enzymes (Curatti and Rubio 2014; López-Torrejón et al. 2016) and the control of enzyme metalation, given the abundance of copper, iron, manganese and zinc in the mitochondrial matrix (Pierrel et al. 2007; Pérez-González et al. 2017). However, one of the challenges involved in mitochondrial targeting is the complex structure of the organelle, which features a double membrane separated by an inner matrix, and folded internal cristae separated by a further membrane, allowing the localization and separation of proteins that require specific environments for their activity (Lill and Mühlenhoff 2008). Nuclear-encoded mitochondrial proteins may therefore feature complex targeting peptides up to 90 amino acids in length carrying the information needed for precise localization within mitochondrial compartments or membranes (Huang et al. 2009b). The pre-sequences comprise different groups of amino acids with distinct physicochemical properties and/or mitochondrial outer membrane recognition motifs (Fukasawa et al. 2015). These properties determine the ultimate destination of native and heterologous proteins within the various spaces and membranes of the mitochondrion (Dudek et al. 2013).

The direct experimental analysis of the plant mitochondrial proteome has been carried out predominantly in dicot species such as Arabidopsis (Kruft et al. 2001; Heazlewood et al. 2004; Lee et al. 2012), tobacco (Huang et al. 1990; Allen et al. 2017) and pea (Bardel et al. 2002). In contrast, only a few reports have described the mitochondrial proteome of monocot species such as rice (Heazlewood et al. 2004; Huang et al. 2009a) and maize (Hochholdinger et al. 2004). In rice, the analysis of 313 nuclear-encoded mitochondrial proteins using four different algorithms revealed that the correct mitochondrial location was predicted in only 60% of cases (Huang et al. 2009a). The number of mitochondrial proteins predicted to be involved in the electron transport chain, tricarboxylic acid cycle and stress responses was well conserved between rice and Arabidopsis (Heazlewood et al. 2004; Huang et al. 2011). The relatively low predictive accuracy of the localization algorithms highlights the importance of experimental validation, particularly when attempting to target recombinant proteins to the mitochondria.

The best way to determine the activity of mitochondrial targeting peptides is to test them using a visible marker, such as enhanced green fluorescent protein (eGFP) adapted for optimal activity in plants (Chiu et al. 1996; Snapp 2005). This will confirm the potential suitability of the import sequence activity, although it does not guarantee the import of other proteins because the structure of the linked protein can also influence the efficiency of targeting peptides (Van Steeg et al. 1986). We therefore generated transgenic rice callus and plants in which nuclear-encoded recombinant eGFP was targeted to the mitochondria using six different mitochondrial N-terminal targeting peptides. These sequences were derived from diverse phylogenetic origins (higher and lower eukaryotes) and varied in their targeting probability scores, motifs and physicochemical properties. We analyzed the expression and localization of eGFP by immunoblot as well as confocal and electron microscopy in order to determine factors responsible for the effectiveness of mitochondrial targeting in rice.

## Materials and methods

### Expression constructs

We selected six different mitochondrial pre-sequences representing different phylogenetic origins. Two of the sequences were from the yeast *Saccharomyces cerevisiae*: the alpha subunit of the F1 sector of the mitochondrial F1F0 ATP synthase protein ATPA (NCBI: NP_009453.2) and the cytochrome oxidase subunit IV protein COX4 (NCBI: NP_011328.1). The third sequence was from the ascomycete fungus *Neurospora crassa*: subunit 9 of the mitochondrial ATPase protein, SU9 (NCBI: XM_954801.3). The other three sequences were from plants. Two were derived from dicot species: the *Nicotiana plumbaginifolia* F1-ATPase from the β-subunit protein MTS2 (NCBI: X02868.1) and the *Arabidopsis thaliana* γ-subunit of the mitochondrial ATP synthase pFA (NCBI: NM_128864.4). The final sequence represented the endogenous rice (*Oryza sativa* ssp. *japonica*) succinyl-CoA synthetase protein β-chain OsSCSb (NCBI: Q6K9N6).

The expression plasmids were generated using the MoClo cloning system (Weber et al. 2011) and an in-house destination vector comprising the pUC57 vector backbone (GenScript, Piscataway, NJ, USA) joined to the cloning cassette of the Level 1 Position 2 MoClo vector pICH47742. The vector was amplified using primers 591 (5′-AAG CCC ACG AAG TGT GGG GTG CCT AAT GAG TGA GCT AAC TCA CA-3′) and 592 (5′-TTA ACA CAG AGT GGC CAG CCC CGA CAC CCG CCA ACA CCC G-3′) and the 2200-bp AdeI digestion product was ligated to the 650-bp fragment released from pICH47742 by digestion with the same enzyme. The recombinant in-house vector (pUC57-L1P2) is available from Addgene (ID: 109221). All the Level 1 Position 2 plasmids were constructed using the strong constitutive maize ubiquitin-1 promoter including the first intron and the nopaline synthase (*nos*) terminator to control eGFP expression.

After MoClo restriction/ligation, 20 µl of the reaction mix was used to transform *Escherichia coli* DH5α competent cells. Positive clones were selected on lysogeny broth (LB) solid medium containing 100 µg/ml ampicillin (Sigma-Aldrich, Darmstadt, Germany), 20 µg/ml X-gal (Duchefa Biochemie, Haarlem, Netherlands) and 1 mM isopropyl β-D-1-thiogalactopyranoside (IPTG; Sigma-Aldrich). Plasmid DNA was extracted using the GenElute Plasmid Miniprep Kit (Sigma-Aldrich). The final constructs were named Ubi:SU9-eGFP-tNos, Ubi:COX4-eGFP-tNos, Ubi:MTS2-eGFP-tNos, Ubi:pFA-eGFP-tNos, Ubi:ATPA-eGFP-tNos and Ubi1:OsSCSb-eGFP-tNos. The integrity of all plasmids was verified by sequencing (Macrogen, Madrid, Spain).

### Transformation of rice callus and regeneration of transgenic plants

The six test vectors containing the individual mitochondrial targeting peptide sequences fused to eGFP were introduced separately into rice embryos, together with the *hpt* gene for selection as described by Christou et al. (1991) and Sudhakar et al. (1998). We selected five representative independent callus lines and corresponding regenerated plants for each construct for all subsequent analyses from among a population of at least 50 independent lines per construct.

### Protein extraction and immunoblot analysis

Total rice protein extracts were prepared by grinding 0.1–0.2 g callus or leaf tissue in liquid nitrogen and thawing the powder in 0.2–0.4 mL of extraction buffer: 20 mM Tris–HCl pH 7.5, 5 mM ethylenediaminetetraacetic acid (EDTA), 0.1% Tween-20, 0.1% sodium dodecylsulfate (SDS), 2 mM phenylmethanesulfonylfluoride (PMSF). The mixture was vortexed for 1 h at 4 °C. Cell debris was removed by centrifugation at 15,000 × *g* for 20 min at 4 °C, and the supernatant was collected and stored at − 80 °C. The protein concentration in the supernatants was determined using the Bradford method (AppliChem, Darmstadt, Germany). We fractionated 80 µg of total rice protein by denaturing SDS-PAGE in polyacrylamide gels containing 10% SDS at 200 V for 60 min, and then electro-transferred the protein to an Immobilon FL polyvinylidene difluoride (PVDF) membrane (Merck, Darmstadt, Germany) using a semidry transfer apparatus (Bio-Rad, Hercules, CA, USA) at 20 V for 45 min. The membrane was immersed in 5% non-fat milk in Tris-buffered saline with Tween-20 (TBST) solution (0.2 M Tris–HCl pH 7.6, 1.37 M NaCl, 0.1% Tween-20) for 1 h at room temperature. Membranes were incubated with anti-eGFP polyclonal antibody SAB4301138 (Sigma-Aldrich) diluted 1:2000 in 5% non-fat milk in TBST overnight at 4 °C, then rinsed three times for 10 min in TBST. The membranes were subsequently incubated with an alkaline phosphatase-conjugated goat anti-rabbit secondary antibody (Sigma-Aldrich) (diluted 1:5000 in 2% non-fat milk in TBS-T) for 1 h at room temperature followed by three 10 min rinses in TBS-T. Signals were detected using SIGMA*FAST* BCIP/NBT tablets (Sigma-Aldrich).

### Confocal microscopy

We used confocal microscopy to confirm the localization of eGFP in small pieces of rice callus (1 mm^3^) or leaf tissue (1 × 10 mm) after incubation with the mitochondrial counterstain Mitotracker Red (Molecular Probes/Invitrogen, Paisley, UK) according to the manufacturer’s recommendations. The tissues were then fixed with 2% paraformaldehyde in 0.1 M sodium phosphate buffer (pH 7.2) and cut into semi-thin sections (30–40 µm) using a CM3050S Research Cryostat (Leica Microsystems, Wetzlar, Germany). The sections were collected on standard glass microscope slides pre-coated with poly-l-lysine and images were captured using an FV1000 laser scanning confocal microscope (Olympus, Hamburg, Germany) with illumination at 488 nm (excitation wavelength of eGFP, multiline argon laser) and 559 nm (excitation wavelength of Mitotracker Red, diode laser). Five different callus lines and leaves (biological replicates) were analyzed per targeting peptide, and the percentage of merged mitochondria (where the green and red color signals co-localized) was counted in a minimum of three images taken from different areas/sections in each sample to determine the mitochondrial targeting efficiency of each peptide.

### Immuno-electron microscopy

Small callus and leaf samples as described above were fixed in 1% glutaraldehyde and 1% paraformaldehyde in 0.1 M sodium phosphate buffer (pH 7.2) for 16–24 h at 4 °C and washed three times (10 min) with the same buffer. After fixation, samples were dehydrated in an ethanol series (30–100%) before embedding in Lowicryl K4 M resin (Polysciences, Hirschberg an der Bergstrasse, Germany) in a cold chamber at − 20 to − 35 °C and inducing polymerization by exposure to ultraviolet light.

Semithin (2 µm) and ultrathin (70–90 nm) sections were prepared using a Reichert–Jung ultra-cut E cryotome (Leica Microsystems). The sections were stained with Richardson’s blue, covered with a drop of DPX slide mounting medium and a coverslip, and observed under a DM4000B microscope (Leica Microsystems). Images were captured using a DFC300 FX 1.4-MP digital color camera equipped with LAS v3.8 (Leica Microsystems). The ultrathin sections were mounted on Formvar carbon-coated gold grids (200 mesh) and incubated for 15 min in blocking buffer for polyclonal antibodies (200 mM Tris–HCl pH 7.4, 1% Tween-20, 0.1% gelatin, 1% BSA) or monoclonal antibodies (10 mM Tris–HCl pH 7.4, 0.9% NaCl, 0.05% PEG 20,000, 3% BSA). The grids were then washed in distilled water and incubated overnight at 4 °C with primary polyclonal anti-eGFP antibody PA5-22688 (Thermo Fisher Scientific, Waltham, MA, USA) diluted 1:200 in blocking buffer, or primary monoclonal anti-eGFP antibody 11814460001 (Sigma-Aldrich) diluted 1:500 in blocking buffer. We cross-adsorbed the polyclonal antibody following the protocol of Deena and Fletcher (1993). After washing in distilled water, followed by a further 30-min incubation in the appropriate blocking buffer and another wash, the grids were incubated at room temperature for 1 h with the 15-nm gold-conjugated secondary antibody diluted 1:20 in the appropriate blocking buffer: goat-anti-rabbit IgG for the polyclonal antibody, or EM-grade goat-anti-mouse IgG for the monoclonal antibody (Electron Microscopy Sciences, Hatfield, PA, USA). Finally, the grids were contrasted with 1% uranyl acetate in water (20 min) and Reynold’s lead citrate (2 min) before observation in an EM 910 Transmission Electron Microscope (Zeiss, Oberkochen, Germany). We analyzed at least two grids per treatment and sample (callus or leaf tissue). More than 10 areas containing mitochondria were registered per treatment.

### Bioinformatics

Targeting peptide cleavage sites, probability scores and physicochemical properties were determined using MitoFates (http://mitf.cbrc.jp/MitoFates/cgibin/top.cgi), MitoProt (https://ihg.gsf.de/ihg/mitoprot.html) and TPpred v3.0 (https://tppred3.biocomp.unibo.it/ tppred3) (Claros and Vincens 1996; Fukasawa et al. 2015; Savojardo et al. 2015).

## Results

### Probability scores and recognition motifs of selected mitochondrial targeting peptides

**Fig. 1:**
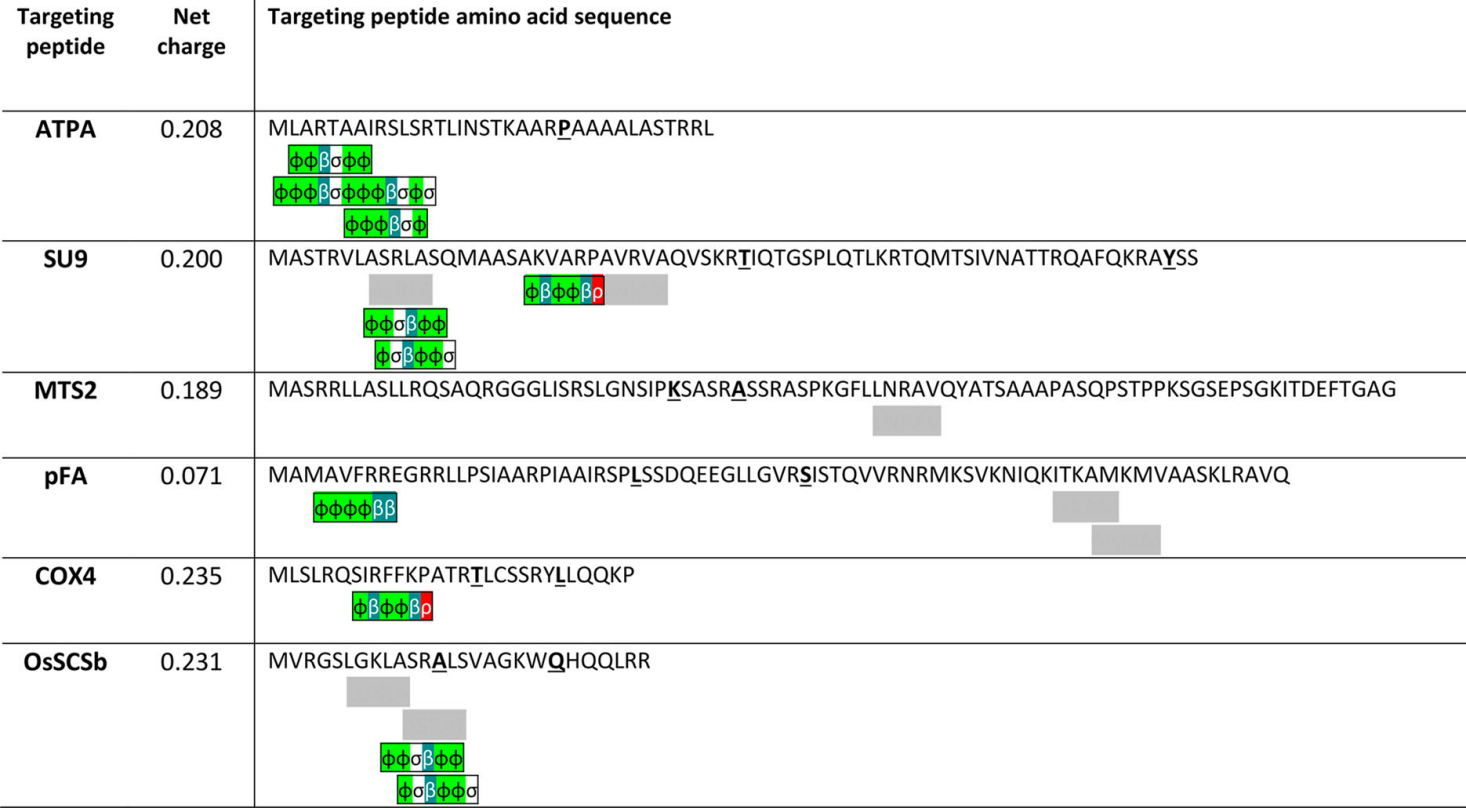
Properties of targeting peptides and the structural relationships among the various motifs. N-terminal hexamer motifs are described using the following symbols to denote groups of amino acids: φ = hydrophobic (L, F, I, V, W, Y, M, C or A), β = basic (R, K or H), α = acidic (E, D), σ = polar (S, T, N or Q), and ρ = secondary structure breaker (P or G). Gray boxes indicate Tom20 recognition motifs. Underlined amino acids are predicted cleavage sites

We selected six mitochondrial targeting peptides for analysis (ATPA, COX4, SU9, MTS2, pFA and OsSCSb), whose properties and phylogenetic origins are summarized in Table 1. Bioinformatics analysis revealed that all six targeting peptides are positively charged and achieve high targeting probability scores but feature different numbers of Tom20 recognition motifs and N-terminal hexamer motifs, as described by Fukasawa et al. (2015). Hexamer motifs are enhancer motifs that interact with hydrophobic binding pockets on the Tom20 receptor and improve mitochondrial targeting effectiveness and accuracy (Obita et al. 2003; Fukasawa et al. 2015). The structural relationships among the various motifs are shown in Fig. 1.

**Table 1:** Characteristics of the six mitochondrial targeting peptides

Peptide	Source	Targeting probability (%)	Size (aa)	Predicted cleavage position (aa)	No. of Tom20 recognition motifs	No. of N-terminal hexamer motifs
ATPA	*Saccharomyces cerevisiae*	99	35	24	0	4
COX4	*Saccharomyces cerevisiae*	92	29	17	0	1
SU9	*Neurospora crassa*	98	69	35/67	2 (ASRLA, AVRVA)	3
MTS2	*Nicotiana plumbaginifolia*	97	87	37/42	1 (LNRAV)	0
pFA	*Arabidopsis thaliana*	97	77	30/42	2 (ITKAM, AMKMV)	1
OsSCSb	*Oryza sativa* ssp*. japonica*	86	27	13	2 (LGKLA, ASRAL)	2

### Expression of eGFP in rice

**Fig. 2:**
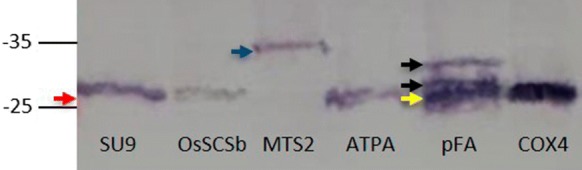
Analysis of six constructs targeting the mitochondria in rice (SU9-eGFP, OsSCSb-eGFP, MTS2-eGFP, ATPA-eGFP, pFA-eGFP and COX4-eGFP) by denaturing SDS-PAGE and western blot. The results represent transformed callus lines but identical profiles were seen in the leaves of transgenic plants. The red arrow (~ 27 kDa) indicates the molecular weight of correctly processed eGFP. The blue arrow (~ 35 kDa) indicates eGFP with a non-cleaved MTS2 peptide. The black arrows (~ 30 and ~ 32 kDa) indicate the products generated following the correct processing of the pFA peptide and the yellow arrow (~ 28 kDa) indicates cleavage closer to the native end of the eGFP

The six mitochondrial targeting peptides we tested included three with single predicted cleavage sites (ATPA, COX4 and OsSCSb) and three with dual predicted cleavage sites (SU9, MTS2 and pFA) based on combined analysis using three bioinformatics programs: MitoFates, MitoProt and TPpred. The six recombinant eGFP constructs were expressed in rice. The callus and leaf extracts were analyzed by immunoblot to determine the size of the recovered eGFP products (Fig. 2). The mature eGFP has a molecular weight of 27 kDa, but all six targeting peptides have internal cleavage sites leaving a remnant on the processed protein, so we anticipated the mature processed products would be somewhat higher in molecular weight than native eGFP, depending on the length of the remnant (Fig. 1). The ATPA, COX4 and OsSCSb variants of eGFP yielded bands of ~ 28 kDa, consistent with successful cleavage of the targeting peptide and the presence of a remnant ranging in length from 11 to 14 residues. The SU9 variant of eGFP yielded a ~ 27 kDa band, consistent with cleavage at position 67 of 69 and the presence of a comparatively small dipeptide remnant. For these four constructs, we therefore observed complete and successful cleavage of the targeting peptide in rice. The pFA sequence has predicted cleavage sites at positions 30 and 42 of 77, which should leave a remnant of at least 35 amino acids. Here we observed three distinct bands of ~ 28, ~ 30 and ~ 32 kDa. The 30 and 32 kDa bands reflected the anticipated sizes of the processed forms of pFA generated by cleavage at the predicted processing sites, whereas the other band indicated that the peptide is also cleaved closer to the native end of the eGFP than expected based on the predictions. Finally, the MTS2 targeting peptide has predicted cleavage sites at positions 37 and 42 of 87, but the ~ 35 kDa band we detected indicated that neither site was cleaved successfully (Fig. 2).

### Localization of eGFP in rice and the effectiveness of the targeting peptides

**Fig. 3:**
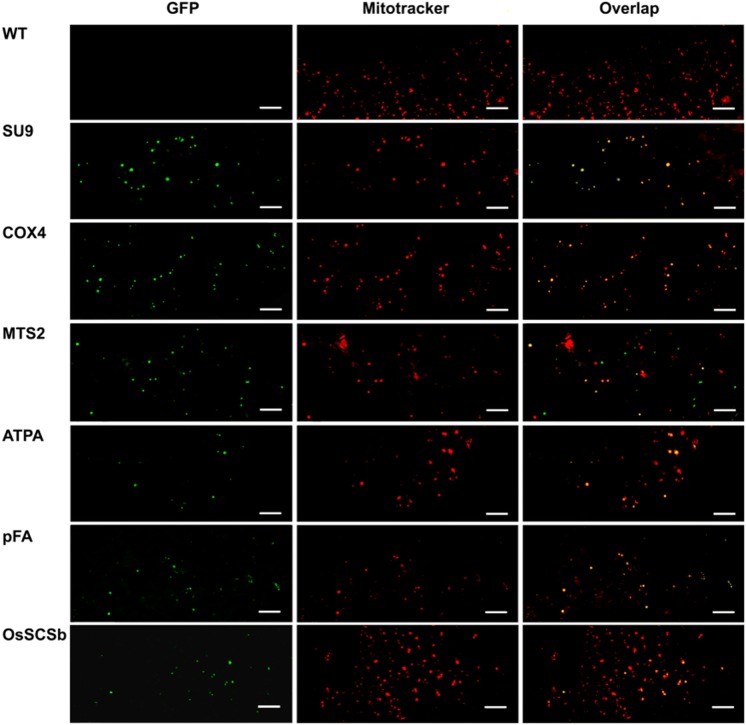
Confocal laser scanning microscopy images of wild-type (WT) rice callus and callus lines transformed with eGFP constructs linked to the SU9, COX4, MTS2, ATPA, pFA and OsSCSb mitochondrial targeting peptides. The three columns show the individual signals for eGFP (green fluorescence) and Mitotracker Red (red fluorescence) and the overlap (merged confocal image mixing green and red fluorescence) (bars = 20 µm). Five different callus lines and leaves (biological replicates) were analyzed per targeting peptide, and the percentage of merged mitochondria (where the green and red color signals co-localized) was counted in a minimum of three images taken from different areas/sections in each sample to determine the mitochrondrial targeting efficiency of each peptide

**Fig. 4:**
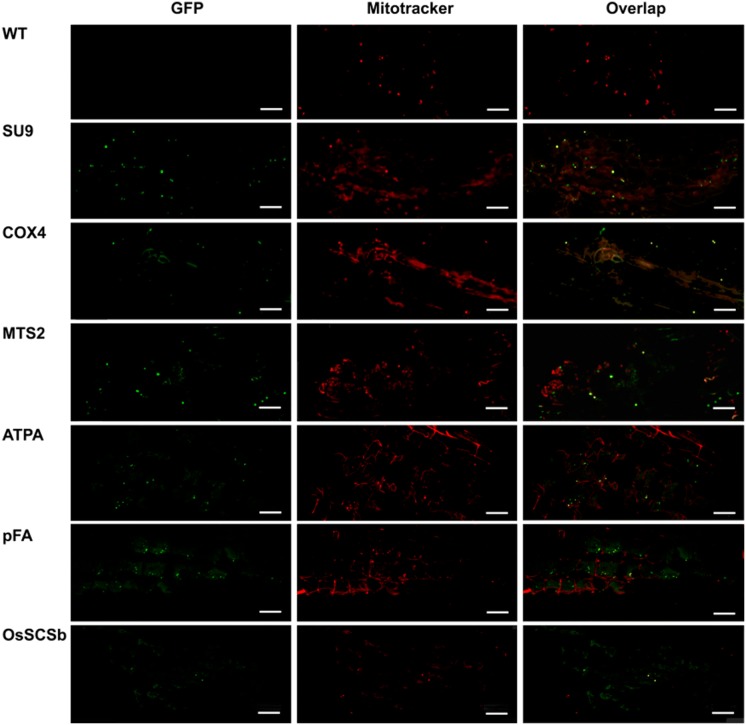
Confocal laser scanning microscopy images of wild-type (WT) rice leaves and leaves from transgenic lines transformed with eGFP constructs linked to the SU9, COX4, MTS2, ATPA, pFA and OsSCSb mitochondrial targeting peptides. The three columns show the individual signals for eGFP (green fluorescence) and Mitotracker Red (red fluorescence) and the overlap (merged confocal image mixing green and red fluorescence) (bars = 20 µm)

**Fig. 5:**
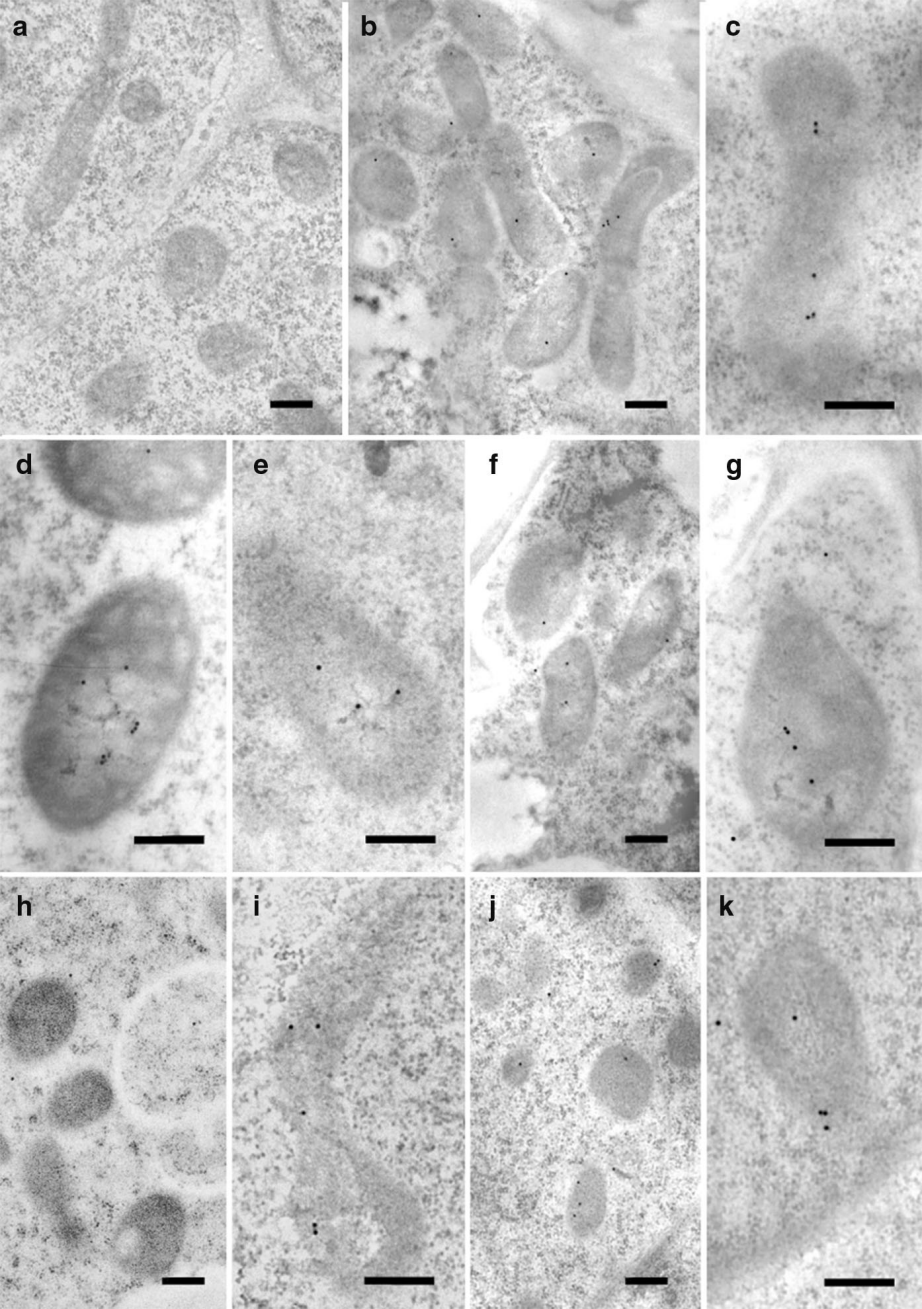
Immunogold labeling of eGFP in the mitochondria of rice callus cells using a GFP-specific monoclonal antibody (diluted 1:200) (**a**–**c**, **f**–**k**) or eGFP polyclonal antibody (diluted 1:500) (**d** = non-cross adsorbed, **e** = cross-adsorbed). Monoclonal antibodies were used to confirm the localization profile revealed by the cross-adsorbed polyclonal antibody although the signal produced by the monoclonal antibody was weaker. **a** Wild-type cells. **b**–**e** SU9-eGFP (**b**, **c**  = monoclonal antibody, **d**, **e** = polyclonal antibody). **f**–**g** Cox4-eGFP is also labeled outside the mitochondria. **h**–**i** MTS2-eGFP is labeled in only a few mitochondria but also in the cytosol. **j**–**k** pFA-eGFP is labeled inside the mitochondria (bars = 200 nm; gold particle size = 15 nm)

**Fig. 6:**
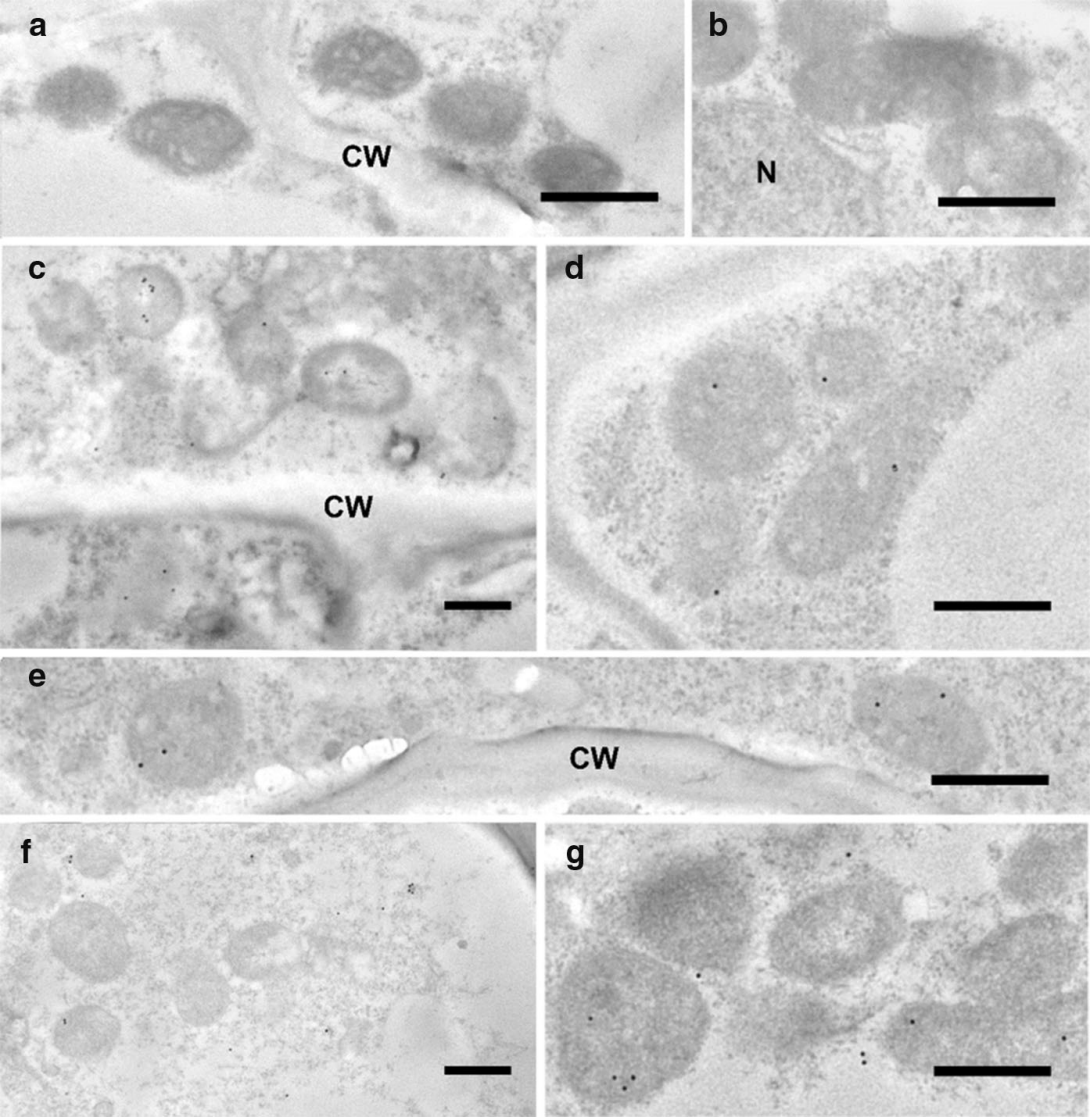
Immunogold labeling of eGFP in the mitochondria of rice leaf cells using a GFP-specific monoclonal antibody (diluted 1:200). **a**–**b** = wild-type, **c**–**d** = SU9-eGFP, **e** = pFA-eGFP, **f** = MTS2-eGFP, **g** = Cox4-eGFP (CW = cell wall, N = nucleus; bars = 500 nm; gold particle size = 15 nm)

**Fig. 7:**
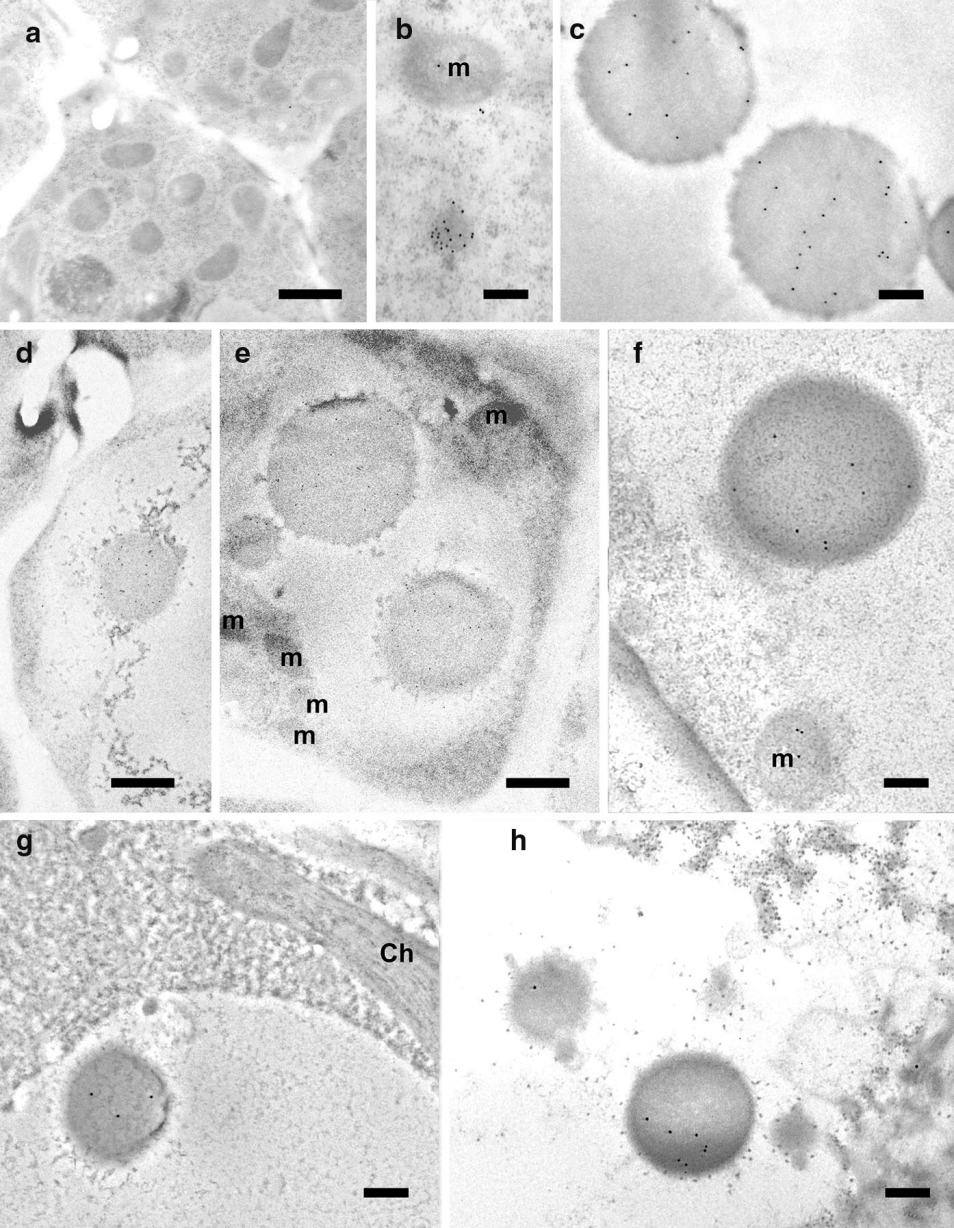
Immunogold labeling of protein bodies in rice callus and leaf cells using a GFP-specific monoclonal antibody (diluted 1:200). **a** Wild-type negative control, showing no protein bodies. **b** Labeled protein body in the cytosol of callus cells expressing Cox4-eGFP. Labeled protein bodies in the vacuole of callus cells (**c**) and leaf cells (**g**–**h**) expressing SU9-eGFP. **d**–**e** Large protein bodies close to (**d**) and inside (**e**) the vacuoles of callus cells expressing MTS2-eGFP. **f** Labeled protein body in callus cells expressing pFA-eGFP (m = mitochondria; bars **a**, **g** and **h** = 1 µm, **b**–**f** = 200 nm; gold particle size = 15 nm)

Having established that five of the six peptides were partially or fully cleaved, we analyzed the localization of eGFP in rice callus (Fig. 3) and in the leaves of the corresponding regenerated plants (Fig. 4) by confocal microscopy. The penetration of the Mitotracker Red marker was more effective in callus than in leaves giving clearer results, but in both cases the green fluorescence of the eGFP and the red fluorescence of the Mitotracker Red marker co-localized in more than 70% of the merged images of tissues transformed with the ATPA, COX4, SU9, pFA and OsSCSb variants of eGFP, whereas the two signals were co-localized in fewer than 15% of the merged images of tissues transformed with the MTS2-eGFP construct. Immunogold labeling was carried out in callus and leaf tissue expressing four of the six constructs (two with the correct, anticipated cleavage patterns as well as pFA with the additional cleavage product and MTS2 which did not show evidence of cleavage). The 15-nm gold particles were strongly associated with the inner mitochondrial membrane and/or mitochondrial matrix in callus (Fig. 5) and leaf (Fig. 6) tissues transformed with the COX4, SU9 and pFA variants, whereas in tissues transformed with the MTS2-eGFP construct the labeling was mostly restricted to the cytosol. For all four targeting peptides, we also observed strongly-labeled protein bodies in the cytosol (Fig. 7), which is consistent with earlier observations of GFP-prolamin fusions in rice (Saito et al. 2009; Shigemitsu et al. 2013). These signals most likely represent eGFP aggregates, which we detected in the vacuoles of callus and leaf tissues expressing each of the four constructs (Fig. 7). The ectopic formation of protein bodies in the leaves is likely to reflect our use of a strong, constitutive promoter (Saberianfar et al. 2016). The aggregates were larger in callus samples expressing MTS2-eGFP compared to those expressing the COX4, SU9 and pFA variants (Supplementary Figs. 1 and 2). Very occasionally, we observed non-specific labeling of the nucleus and chloroplasts in tissues expressing each of the four constructs (Supplementary Figs. 3 and 4).

## Discussion

Proteins targeted for mitochondrial import may be directed to the inner matrix, the inner or outer membranes, or the intermembrane space (Chacinska et al. 2009; Weis et al. 2013). Most mitochondrial proteins are encoded by the nuclear genome, synthesized on cytosolic ribosomes and ultimately translocated to the appropriate sub-mitochondrial location (Taylor and Pfanner 2004; Dolezal et al. 2006). The mechanism that controls protein import into mitochondria involves an N-terminal pre-sequence or targeting peptide which directs the protein to the correct internal location, and the peptide is removed by proteolytic cleavage when the protein reaches its destination (Huang et al. 2009a; Carrie et al. 2015).

Mitochondrial targeting peptides tend to feature a high content of hydrophobic and positively charged amino acid residues, a near absence of negatively charged residues, and a very low abundance of acidic amino acids (Berglund et al. 2009). Mitochondrial pre-sequences have the tendency to form an amphiphilic α-helix, which interacts with the import receptor Tom20 (Pfanner and Geissler 2001; Endo and Kohda 2002), a 20-kDa subunit of the outer mitochondrial membrane complex translocase (Saitoh et al. 2007). Tom20 recognizes the same motifs in plants and yeast, conventionally represented as φxxφφ (where φ is a hydrophobic amino acid and x is any amino acid). However, there are significant differences in the topology, localization, amino acid variations, binding motifs and number of Tom20 proteins in different organisms (Obita et al. 2003; Saitoh et al. 2007).

The Tom20 protein in fungi and animals is anchored via its N-terminus to the mitochondrial outer membrane and therefore exposes its C-terminal domain to the cytosol. In contrast, Tom20 in plants is anchored to mitochondria via its C-terminus and the N-terminal domain is exposed. Accordingly, plant Tom20 proteins are not orthologous to those of fungi and animals, and plant mitochondria also lack the other two receptor components that have been functionally characterized in yeast and mammalian systems, namely Tom70 and Tom22 (Macasev et al. 2004; Lister et al. 2007; Huang et al. 2009a; Rimmer et al. 2011). Previous studies have shown that plant Tom20 proteins interact with the amino acid domains LRTLA and LRRFV in rice superoxide dismutase and Arabidopsis threonyl tRNA synthetase (Zhang et al. 2010). Even though Tom20 recognition motifs have been well characterized, they are not essential for mitochondrial protein import (Mukhopadhyay et al. 2006; Lister et al. 2007; Lee et al. 2012). For example, when the Tom20 recognition site of pFA was deleted, this reduced the efficiency of mitochondrial import in Arabidopsis by only 20% (Lee et al. 2012). However, there are four Tom20 paralogs in the Arabidopsis genome, three of which are known to be functional, whereas rice (like yeast) has only a single Tom20 gene (Lister et al. 2007). Despite the major differences between Tom20 proteins in plants and fungi, mitochondrial targeting peptides from yeast and filamentous fungi are functional in plants. We tested three such peptides, namely ATPA and COX4 from *S. cerevisiae* and SU9 from *N. crassa*. Previously, all three peptides have been shown to successfully direct recombinant proteins to mitochondria in yeast, tobacco and Arabidopsis (Nelson et al. 2007; Burén et al. 2017; Pérez-González et al. 2017) and similarly we found that all three were able to direct eGFP to the mitochondria in rice. Interestingly, only one of these peptides features a Tom20-recognition motif but all three contain N-terminal hexamers. This provides more evidence that Tom20-recognition motifs are not strictly required for mitochondrial import in plants.

We also tested three plant-derived targeting peptides, including the Arabidopsis pFA sequence which was mentioned briefly above. The others were the OsSCSb peptide from rice and the MTS2 sequence from *N. plumbaginifolia*. Lee et al. (2012) showed that Arabidopsis pFA contains multiple sequence motifs that target different mitochondrial compartments. In addition to the IAARP Tom20-recognition motif, the sequences DQEEG and VVRNR are involved in translocation across the mitochondrial membrane, and the sequences RLLPS and SISTQ pull proteins into the matrix. In another study, the Arabidopsis pFA peptide efficiently imported 16 nitrogenase protein components into the mitochondrial matrix of *Nicotiana benthamiana* in transient expression experiments, as well as a GFP fusion protein. A ~ 30 kDa band was observed corresponding to the correctly processed GFP as well as a less abundant ~ 28 kDa band representing an alternatively processed form or a degradation product (Allen et al. 2017). Similarly, we found that the Arabidopsis pFA targeting peptide was sufficient for the mitochondrial import of eGFP in rice, yielding three different bands of ~ 28, ~ 30 and ~ 32 kDa. Our bioinformatics analysis suggested that the 30 and 32 kDa bands represented the expected processed forms of the pFA-eGFP fusion and the remaining band was an alternatively processed form or a degradation product. This strongly suggests that the pFA peptide works as effectively in rice (this study) as it does in dicot species (Lee et al. 2012; Allen et al. 2017). We tested the OsSCSb peptide based on an earlier report with tentative bioinformatics and mass spectrometry data predicting its mitochondrial targeting ability (Huang et al. 2009b). Our empirical data supported this prediction by confirming the mitochondrial import of eGFP, the first time this sequence has been shown directly to function as a mitochondrial import peptide. Notably, both pFA and OsSCSb contain Tom20-binding motifs and N-terminal hexamers.

The *N. plumbaginifolia* MTS2 sequence was one of the first mitochondrial targeting peptides to be characterized in plants and has been shown to work in tobacco, although the authors did not present detailed microscopic analysis (Boutry and Chua 1985; Chaumont et al. 1994). Surprisingly, we found that the MTS2 sequence appeared insufficient for the mitochondrial import of eGFP in rice, and immunoblot experiments provided evidence that the protein was not cleaved in rice cells. Among the six peptides we tested, MTS2 was the only one that contained at least one Tom20-binding motif but no N-terminal hexamers, indicating that the latter may be necessary for mitochondrial import in rice. Similarly, mutations in the N-terminal hexamers of potato formate dehydrogenase were previously shown to block the mitochondrial import of GFP fusion proteins (Ambard-Bretteville et al. 2003). In contrast, Gnanasambandam et al. (2008) showed in a transient expression system that the MTS2 peptide efficiently targeted GFP to mitochondria in several plants: five monocots (sugarcane, wheat, corn, sorghum and onion) and seven dicots (cucumber, cauliflower, tomato, capsicum, pumpkin, coriander and sunflower). The MTS2 peptide therefore appears to be strongly influenced by the choice of cargo protein and host species, and detailed microscopy should be carried out to confirm the correct targeting of recombinant proteins using this sequence.

Confocal microscopy alone may not be sufficient to draw definitive conclusions about the efficiency or accuracy of targeting peptides in stably transformed intact plants. Our confocal microscopy data indicated efficient mitochondrial import in rice callus and leaf tissues, but detailed analysis by immuno-electron microscopy revealed a certain level of non-specific labeling in the cytoplasm (Fig. 7). This may reflect the formation of protein body-like structures in response to the high levels of recombinant protein synthesis, as discussed by Gutierrez et al. (2013). Many previous studies have reported that protein bodies can be induced in rice and wheat callus and seeds (Arcalis et al. 2004; Saito et al. 2009; Takaiwa et al. 2009; Shigemitsu et al. 2013). Protein bodies do not usually form naturally in leaves, but they can be induced by the high-level expression of recombinant proteins, for example in *N. benthamiana* (Saberianfar et al. 2016). Protein bodies form mainly in the endoplasmic reticulum but may bud off into the cytosol as unattached organelles or may be removed to the vacuole (Levanony et al. 1992). In wheat, storage proteins aggregate into protein bodies and are then transported to the vacuoles without passing through the Golgi complex (Levanony et al. 1992).

We also observed non-specific labeling in the nucleus (Supplementary Fig. 3) and in the chloroplasts (Supplementary Fig. 4). The non-specific labeling in the nucleus is likely to be genuine, and probably occurs because eGFP is small enough (~ 27 kDa) to enter the nucleus by free bidirectional diffusion through the nuclear pore complex, which has a size exclusion limit of 40–60 kDa (Köhler et al. 1997; Wei et al. 2003; Seibel et al. 2007). The non-specific labeling in the chloroplast is more likely to be an experimental artifact. Berglund et al. (2009) previously reported that mitochondrial and chloroplast targeting peptides are similar enough that mitochondrial proteins can be inadvertently directed to the plastids. However, we found that the erroneous labeling of plastids could be eliminated by cross-adsorbing the polyclonal antibody with wild-type leaf extract (Supplementary Fig. 4) suggesting that the fidelity of mitochondrial and plastid targeting in vivo is preserved.

## Conclusions

Mitochondrial protein import is a complex, multistep process including the recognition of targeting peptides before translocation and peptide cleavage. N-terminal hexamer motifs and Tom20-recognition motifs are thought to enhance mitochondrial targeting efficiency and accuracy. However, detailed analysis of the sequence motifs in each targeting peptide is necessary to understand the interactions between targeting peptides and translocator components during protein import. Here we demonstrated that the mitochondrial import of nuclear-encoded eGFP in rice requires only a single N-terminal hexamer motif whereas the Tom20-binding motifs are not strictly required, at least based on the evidence from the six targeting peptides we tested. The only one of six targeting peptides that did not function in rice in our experiments features a single Tom20-binding motif but lacks an N-terminal hexamer. The efficiency of mitochondrial import in rice is difficult to quantify but can be determined by combining stable gene expression with immunogold labeling to investigate in detail the subcellular localization of proteins targeted for mitochondrial import. Having tested a range of targeting peptides from fungi and plants, we conclude that the presence of particular functional motifs, specifically the N-terminal hexamer, is likely to be more important for successful mitochondrial import in rice than the phylogenetic origin of the targeting peptide.

## Electronic supplementary material

Below is the link to the electronic supplementary material.

Supplementary Fig. 1 Confocal and light microscopy images of callus cells expressing MTS2-eGFP. A. Confocal laser scanning microscopy image, with arrows highlighting the mitochondria. B. Light microscopy image. C-D. Immuno-electron microscopy images showing the detection of eGFP using a polyclonal antibody (diluted 1:500) (m = mitochondria, pb = protein body; bars A-B = 20 µm, C-D = 2 µm; gold particle size = 15 nm) (TIFF 1827 kb)

Supplementary Fig. 2 Light microscopy images of transformed rice callus. Semithin sections (2 µm) of (A) wild-type negative control callus showing no protein bodies, or callus expressing (B–C) SU9-eGFP, (D) Cox4-eGFP, (E) MTS2-eGFP, and (F) pFA-eGFP. Arrows show spherical bodies in the vacuoles. Note that size and quantity appear larger in cells close to the border (B, E) and disrupted cells (E, F). Most of the bodies are lighter blue than the darker nucleoli. The bodies are particularly large in cells expressing MTS2-eGFP (bars = 20 µm) (TIFF 25056 kb)

Supplementary Fig. 3 Immunogold labeling of the nucleus (N) in rice callus cells using a GFP-specific monoclonal antibody (diluted 1:200). Note the specific labeling of mitochondria (m) and the nucleus/nucleolus in A (SU9-eGFP), B (Cox4-eGFP) and C (pFA-eGFP) (bars = 500 nm; gold particle size = 15 nm) (TIFF 3024 kb)

Supplementary Fig. 4 Non-specific immunogold labeling of chloroplasts in rice leaf cells using eGFP polyclonal cross-adsorbed and non-cross-adsorbed antibodies (diluted 1:500). A. Wild-type leaf cells treated with the non-cross-adsorbed antibody (1:500) show the labeling of chloroplasts. B. Wild-type leaf cells treated with the cross-adsorbed antibody showing no labeling of chloroplasts. C. Leaf cells expressing SU9-eGFP treated with the non-cross-adsorbed antibody (1:500). D. Leaf cells expressing SU9-eGFP treated with the cross-adsorbed antibody (bars = 500 nm; gold particle size = 15 nm) (TIFF 2769 kb)
