# Supplementary figures and images for: Recognition motifs rather than phylogenetic origin influence the ability of targeting peptides to import nuclear-encoded recombinant proteins into rice mitochondria
Source: Transgenic Res. 2019 Oct 10;29(1):37–52. doi: 10.1007/s11248-019-00176-9 (PMC7000509; doi:10.1007/s11248-019-00176-9)

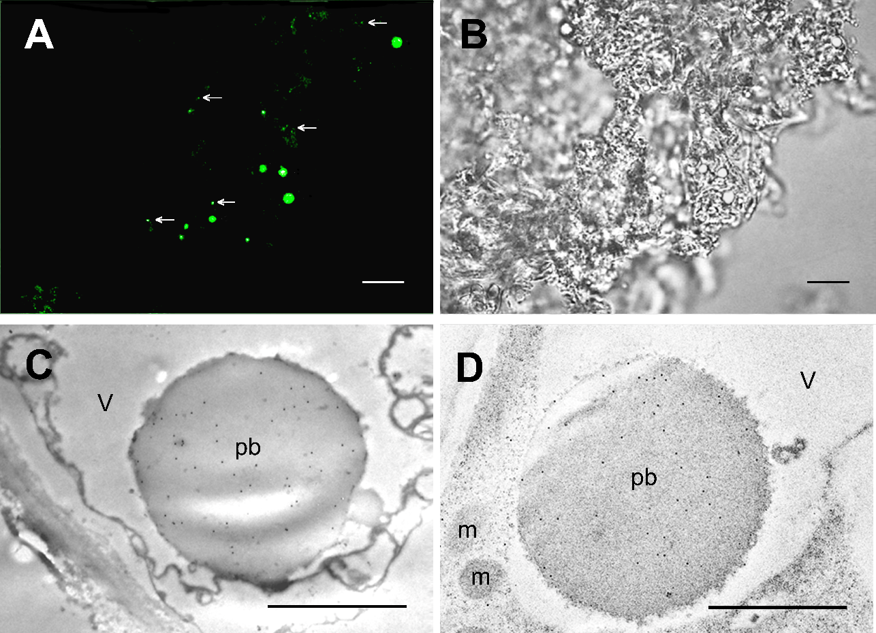

Supplement: Supplementary file 1 — Confocal and light microscopy images of callus cells expressing MTS2-eGFP. A. Confocal laser scanning microscopy image, with arrows highlighting the mitochondria. B. Light microscopy image. C-D. Immuno-electron microscopy images showing the detection of eGFP using a polyclonal antibody (diluted 1:500) (m = mitochondria, pb = protein body; bars A-B = 20 µm, C-D = 2 µm; gold particle size = 15 nm) (TIFF 1827 kb) [file 11248_2019_176_MOESM1_ESM.tif]

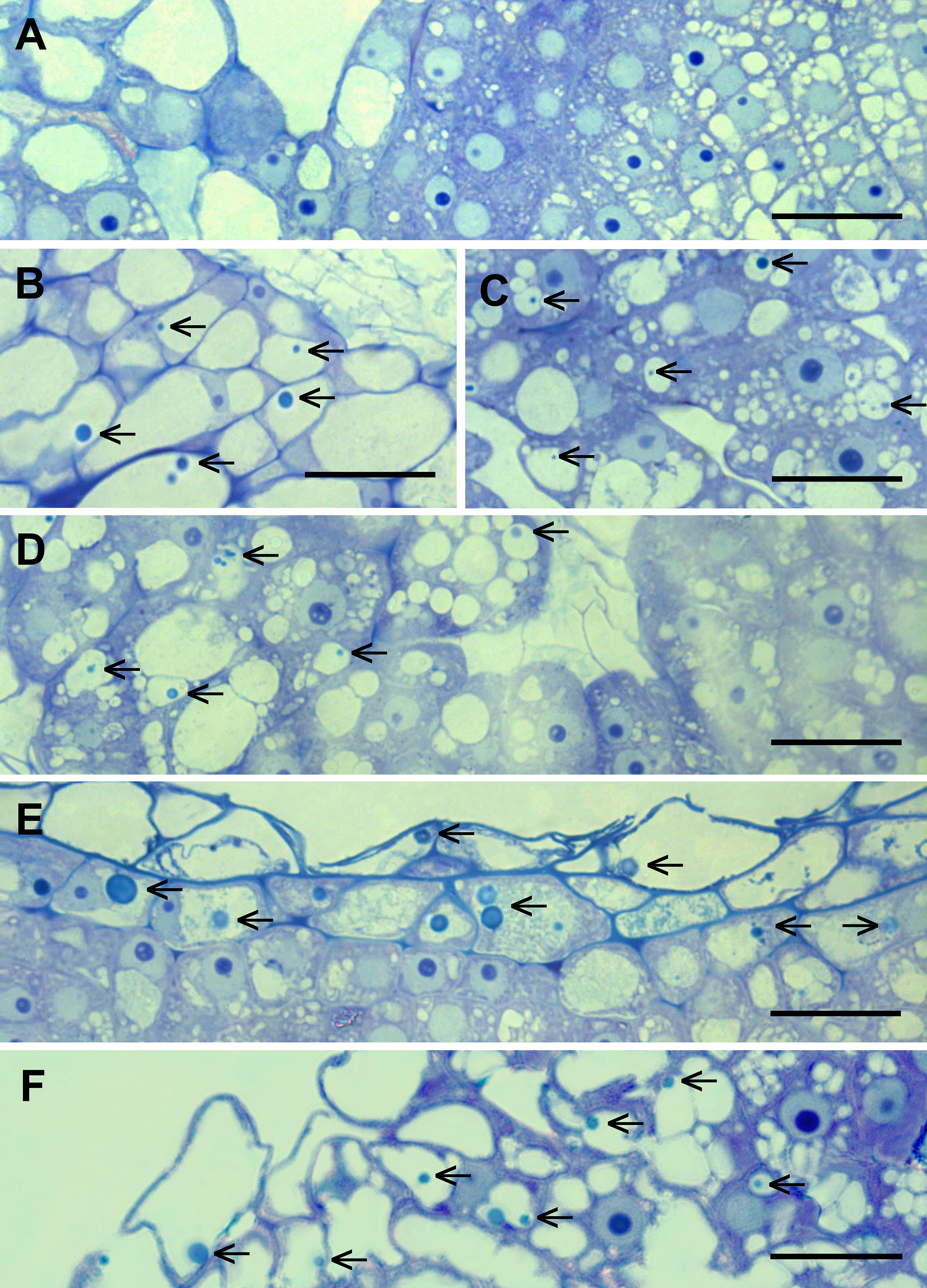

Supplement: Supplementary file 2 — Light microscopy images of transformed rice callus. Semithin sections (2 µm) of (A) wild-type negative control callus showing no protein bodies, or callus expressing (B–C) SU9-eGFP, (D) Cox4-eGFP, (E) MTS2-eGFP, and (F) pFA-eGFP. Arrows show spherical bodies in the vacuoles. Note that size and quantity appear larger in cells close to the border (B, E) and disrupted cells (E, F). Most of the bodies are lighter blue than the darker nucleoli. The bodies are particularly large in cells expressing MTS2-eGFP (bars = 20 µm) (TIFF 25056 kb) [file 11248_2019_176_MOESM2_ESM.tif]

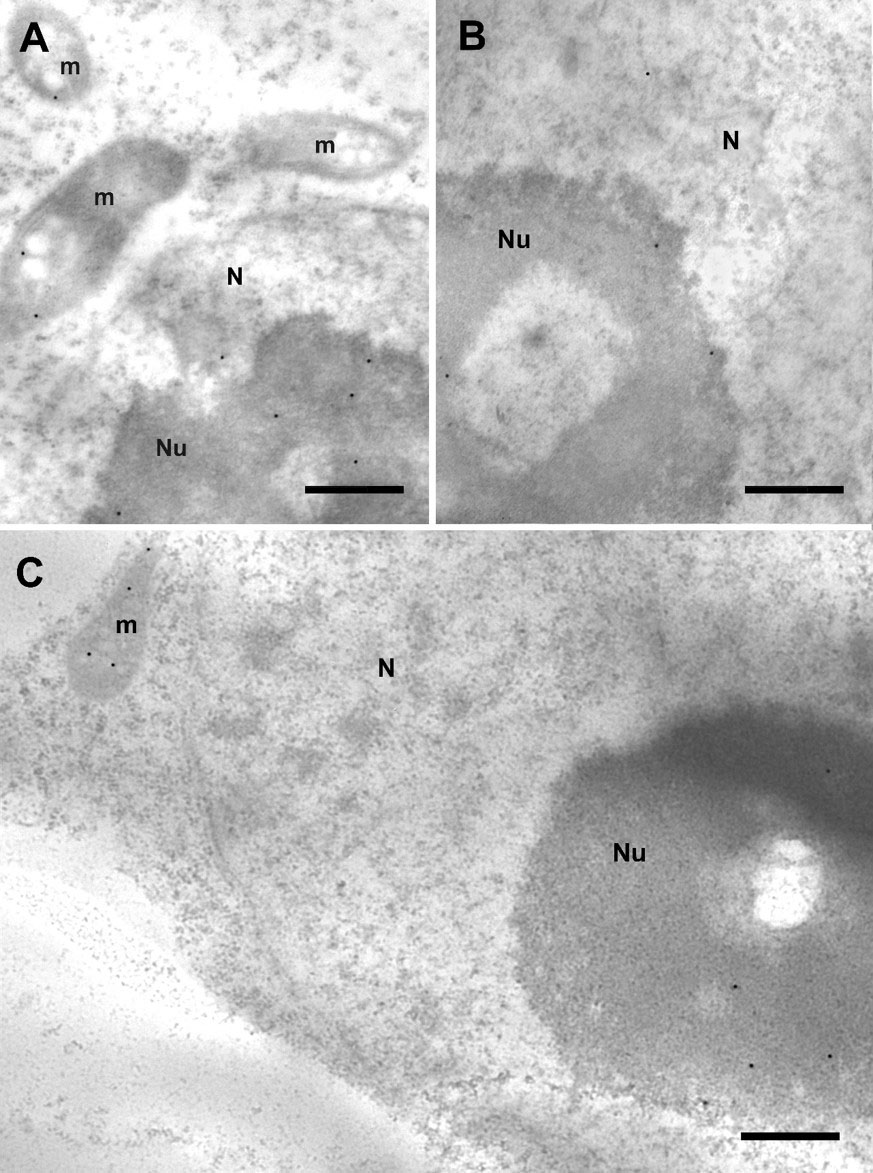

Supplement: Supplementary file 3 — Immunogold labeling of the nucleus (N) in rice callus cells using a GFP-specific monoclonal antibody (diluted 1:200). Note the specific labeling of mitochondria (m) and the nucleus/nucleolus in A (SU9-eGFP), B (Cox4-eGFP) and C (pFA-eGFP) (bars = 500 nm; gold particle size = 15 nm) (TIFF 3024 kb) [file 11248_2019_176_MOESM3_ESM.tif]

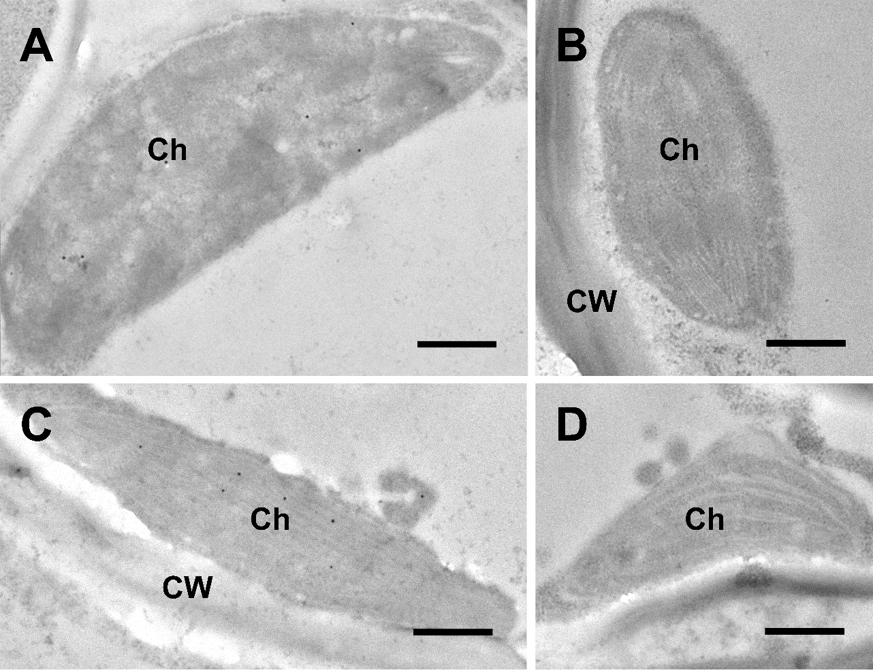

Supplement: Supplementary file 4 — Non-specific immunogold labeling of chloroplasts in rice leaf cells using eGFP polyclonal cross-adsorbed and non-cross-adsorbed antibodies (diluted 1:500). A. Wild-type leaf cells treated with the non-cross-adsorbed antibody (1:500) show the labeling of chloroplasts. B. Wild-type leaf cells treated with the cross-adsorbed antibody showing no labeling of chloroplasts. C. Leaf cells expressing SU9-eGFP treated with the non-cross-adsorbed antibody (1:500). D. Leaf cells expressing SU9-eGFP treated with the cross-adsorbed antibody (bars = 500 nm; gold particle size = 15 nm) (TIFF 2769 kb) [file 11248_2019_176_MOESM4_ESM.tif]
